# Application of Accelerated Predictive Stability Studies in Extemporaneously Compounded Formulations of Chlorhexidine to Assess the Shelf Life

**DOI:** 10.3390/molecules28237925

**Published:** 2023-12-04

**Authors:** Olga González-González, M. Paloma Ballesteros, Juan J. Torrado, Dolores R. Serrano

**Affiliations:** 1 Departamento de Farmacia Galénica y Tecnología Alimentaria, Facultad de Farmacia, Univsersidad Complutense de Madrid, Plaza Ramón y Cajal, s/n, 28040 Madrid, Spain; olgagonzalez@ucm.es (O.G.-G.); pballesp@ucm.es (M.P.B.); 2 Instituto Universitario de Farmacia Industrial (IUFI), Facultad de Farmacia, Universidad Complutense de Madrid, Plaza Ramón y Cajal, s/n, 28040 Madrid, Spain

**Keywords:** chlorhexidine, stability, extemporaneous compounding, degradation

## Abstract

Industrially fabricated medicines have a well-defined shelf life supported by rigorous studies before their approval for commercialization. However, the shelf life of extemporaneous compounding topical formulations prepared at hospitals tends to be shorter, especially when no data are available to prove a longer stability period. Also, the storage conditions are unknown in many circumstances. Accelerated Predictive Stability (APS) studies have been shown to be a useful tool to predict in a faster and more accurate manner the chemical stability of extemporaneously compounded formulations requiring a minimum amount of formulation, thereby reducing the chemical drug waste per study. Shelf life will be allocated based on scientific data without compromising drug efficacy or safety. In this work, the APS approach was applied to the commercially available Cristalmina^®^ (CR) and an extemporaneously compounded formulation of chlorhexidine (DCHX). A different degradation kinetic was found between DCHX and CR (Avrami vs. zero-order kinetics, respectively). This can explain the different shelf life described by the International Council for Harmonisation of Technical Requirements Registration Pharmaceuticals Human Use (ICH) conditions between both formulations. A predicted stability for the DCHX solution was obtained from the extrapolation of the degradation rate in long-term conditions from the Arrhenius equation. The estimated degradation from the Arrhenius equation for DCHX at 5 °C, 25 °C, and 30 °C at 365 days was 3.1%, 17.4%, and 25.9%, respectively. The predicted shelf life, in which the DCHX content was above 90%, was 26.67 months under refrigerated conditions and 5.75 and 2.24 months at 25 and 30 °C, respectively. Currently, the Spanish National Formulary recommends a shelf life of no longer than 3 months at room temperature for DCHX solution. Based on the predicted APS and confirmed by experimental long-term studies, we have demonstrated that the shelf life of DCHX extemporaneously compounded formulations could be prolonged by up to 6 months.

## 1. Introduction

Stability plays a major role in pharmaceutical products as well as in extemporaneously compounded formulations. Industrially fabricated medicines have a well-defined shelf life supported by rigorous studies before their approval for commercialization. However, the shelf life of extemporaneously compounded formulations prepared at hospitals tends to be shorter, especially when no data are available to prove a longer stability period. Also, the storage conditions are unknown in many circumstances, and this results in a waste of medicines that could be prevented if further stability studies are performed that can demonstrate the stability of the formulations over longer periods, especially with topical products.

Additionally, stability studies according to the ICH guidelines are tedious, require a prolonged period of at least six to twelve months, and are costly. Based on these premises, APS studies could be a useful tool to predict the chemical stability of extemporaneously compounded formulations in a less time-consuming manner [1]. APS studies are designed to speed up the degradation rate and predict the kinetic model of degradation, the degradation products, and the suitable storage conditions. In contrast to ICH stability studies, APS studies are performed over a 3–4-week period, combining extreme temperatures ranging from 50 to 80 °C and a variable relative humidity between 10 and 75% [2,3,4]. In APS studies, the isoconversion time is calculated, which is defined as the time to edge of failure, which means the time to reach a certain specification limit for potency or degradation [5,6]. However, the degradation is fixed at certain time points, commonly 3, 6, 12, 18, and 24 months, and the degradation rates are different at each condition in the ICH stability studies, in which the long-term testing is usually performed over a minimum of 12 months at 25 °C ± 2 °C/60% RH ± 5% RH or 30 °C ± 2 °C/65% RH ± 5% RH [1].

The stability prediction in APS studies is based on the Arrhenius equation, which describes the relationship between the rate of degradation and the temperature in a liquid state. Thus, the lower the activation energy, the lesser the energy required to trigger the degradation, and hence, the lower the chemical stability. From the logarithmic form of the Arrhenius equation, the degradation rate becomes a straight line, and in consequence, the degradation rate at mild conditions (25 °C) can be extrapolated [7,8].

To the best of our knowledge, the application of APS studies has only been performed in industrially fabricated medicines. However, the hypothesis underpinning this work is that APS studies can be potentially used to determine the stability of extemporaneously compounding formulations, providing a better understanding of the most suitable storage conditions, packaging materials, and shelf life. In this work, we are applying the APS methodology to compare the stability of chlorhexidine from industrially fabricated medicines (Cristalmina^®^, CR, Madrid, Spain) and extemporaneous topical compounding formulations (chlorhexidine aqueous solution at 0.1%, DCHX) commonly described in the Spanish National Formulary, which are prescribed by clinicians. Long-term and accelerated predictive stability studies were performed, and data were statistically compared. The effect of several packaging materials on the extemporaneously compounded formulations was also investigated under different storage conditions.

## 2. Results

### 2.1. APS Study

The degradation constant (*K*) was calculated under the different conditions in which the formulations were stored. In Table 1, the *K* values obtained from the slope of the different kinetic models tested at each temperature are illustrated. The model with the greatest averaged coefficient of determination (*R*^2^) was considered the most suitable one to predict the *Ea* from the Arrhenius equation. Surprisingly, the DCHX extemporaneous formulation and commercially available CR showed different degradation kinetics. The best-fitting kinetic model of degradation was the Avrami model for the DCHX extemporaneous preparations and the zero-order model for the industrially fabricated medicines of CR (Table 2).

The degradation constant using either the Avrami or zero-order reaction for DCHX and CR, respectively, was employed to extrapolate *Ea* from the Arrhenius equation. In both cases, *R*^2^ was above 0.9, indicating a good fit of the experimental data to the equation. The activation energy for CR was greater (23.3%) than for the DCHX extemporaneously compounded formulations, indicating better chemical stability, as higher energy is required to start the degradation process.

### 2.2. Long-Term Study

**Figure 1:**
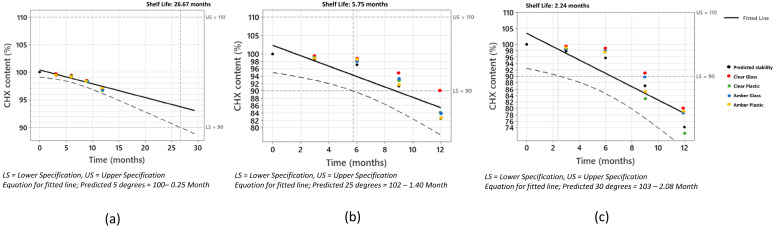
Correlation between the predicted DCHX content and the experimental results. (**a**) Correlation values at 5 °C, (**b**) correlation values at 25 °C, and (**c**) correlation values at 30 °C.

DCHX preparations were kept in different storage conditions, and four different packaging materials for bottles were analyzed at different time points over one year (3, 6, 9, and 12 months) to measure their chemical stability over time and the appearance of degradation products such as p-chloroaniline (PCA). In Figure 1, the drug degradation under different long-term conditions (5, 25, and 30 °C) over time is illustrated and compared with the predicted stability extrapolated from the Arrhenius equation. The experimental results were corrected based on the evaporation rate observed from each container under each condition (Table S1 in Supporting information). This is a key point to bear in mind, as all preparations are aqueous and so are subjected to evaporation, especially at higher temperatures. The estimated degradation from the Arrhenius equation for DCHX at 5 °C, 25 °C, and 30 °C at 365 days was 3.1%, 17.4%, and 25.9%, respectively. The predicted shelf life for which the DCHX content was above 90% was 26.67 months under refrigerated conditions and 5.75 and 2.24 months at 25 and 30 °C, respectively. These results are aligned with the shelf life suggested by the Spanish National Formulary, which recommends no longer than 3 months of stability at room temperature.

Prediction models showed good accuracy with the experimental values at all three tested conditions. The deviation from the experimental real data was larger at 180 and 365 days. Overall, the models (at 25 and 30 °C) tend to be conservative, which means a larger predicted degradation is observed compared to the real one. This can be considered acceptable from an industrial point of view, as the likelihood of failure during long-term stability tests will be lower with this type of predictive model, contrary to those that do not detect that degradation is occurring. One reason behind this deviation is the fact that the APS study was performed using clear glass HPLC vials, while the long-term stability data were obtained using a range of different containers susceptible to different water permeabilities, which will be discussed in the next section.

### 2.3. Container Permeability Study

Water permeability has a crucial effect when performing drug stability studies. The headspace was kept constant in all the containers, as all of them had a 60 mL capacity containing 30 mL of the DCHX solution. The relative humidity of the laboratory where the experiments were performed was close to 30%, and hence, there is a constant tendency for the molecules in the vapor state in the headspace of the containers to equilibrate with the vapor water molecules of the environment. This triggers a constant evaporation rate from the container, which translates into an increase in the concentration of DCHX over time. It is important to quantify the evaporation rate at different time points to correct the DCHX degradation experimental data with the theoretical concentration left at each time point. Glass containers were less susceptible to water permeability compared to plastic containers (Table 3). Also, at greater temperatures, larger evaporation rates were found. Even though a correction factor was applied, this can explain why, especially at 365 days, the experimental degradation of DCHX was lower than the predicted one at 25 and 30 °C.

## 3. Discussion

In this manuscript, for the first time, the applicability of APS studies to calculate the shelf life of extemporaneous liquid compounding formulations has been demonstrated. Pharmaceutical National Agencies tend to be conservative when dictating the shelf life of extemporaneously compounded formulations, as in most cases, there is not a strong scientific background to support this decision [9]. Scientific evidence can extend the shelf life of extemporaneously compounded formulations, reducing drug waste [10]. However, this leads to a waste of medicines in many situations. In the case of DCHX solutions, a shelf life of three months is proposed by the Spanish National Formulary. However, the experimental shelf life of DCHX is over two years when stored under refrigerated conditions or up to almost 6 months at 25 °C, considering as a specification criterion a DCHX content above 90%. Microbiological contamination can be an issue with certain formulations and limit their shelf life, such as eye drops; nevertheless, DCHX is an antimicrobial agent for topical applications and should not suffer from this.

The activation energy predicted in the APS studies was between 18.5 and 24 Kcal/mol, which is in agreement with the activation energy described by the PhD thesis entitled “Aspects of chlorhexidine degradation” (69.5 to 96.1 kJ/mol, equivalent to 16.5–22.9 Kcal/mol) [11]. Surprisingly, the degradation kinetic model was different from the extemporaneously compounded formulation and the industrial one. DCHX followed an Avrami kinetic model, while the CR degradation profile fitted better to a zero-order model. Even though there is no full consensus in the literature about the degradation pathway of DCHX, it seems that DCHX suffers from hydrolysis in aqueous solutions, with the direct formation of PCA being the major pathway in acidic conditions, whereas the indirect formation of PCA via the formation of p-chlorophenylurea is the main pathway in alkaline conditions [12,13]. PCA was not used for quantification purposes as it behaved as an intermediate product that degraded successively into other degradants. Hence, it was difficult to quantify the overall degradation of DCHX and establish a direct correlation using a PCA percentage. The pH of the DCHX and CR solutions ranged between 6 and 6.5, which can result in the mixed direct and indirect formation of PCA. Zero-order kinetics are those in which the degradation rate is unchanged as the amount of drug substance decreases. Nevertheless, most zero-order reactions are pseudo-first or second-order reactions concerning the reactant, in which the reaction is stopped before the degradation rate starts to slow down due to the consumption of the drug [7]. Taking into account that the specification limit for DCHX loss was 10%, greater percentages of degradation are not relevant, and it can be assumed that CR degrades in an apparently linear fashion [7]. However, greater percentages of degradation over time were found for the DCHX solution in the APS studies. Initially, the degradation rate was slow (following zero-order kinetics similar to CR), but over time, the degradation of DCHX increased exponentially, which can be explained by the fact that the degradant products accelerated the degradation kinetics. In conclusion, a complex chemical degradation mechanism is responsible for DCHX following a non-linear degradation (Avrami), in which many consecutive, competing, and reversible kinetics are involved.

APS studies have demonstrated that they can predict the shelf life of extemporaneous formulations reasonably well [14]. A good match between predicted and experimental values was found up to six months when using APS studies. However, due to evaporation issues, the prediction capacity was poorer after longer periods. The APS studies were performed in HPLC glass vials, which can affect their direct comparison with long-term stability studies that employ a different type of container. Novel industrial software utilized by pharmaceutical companies to conduct prediction stability studies, such as ASAP Prime v. 6.0.3 Freethink Technologies, Branford, CT, USA, uses the same principle described in this manuscript, the Arrhenius equation, to calculate the activation energy at extreme conditions, followed by extrapolation under ICH conditions. To avoid this issue, ASAP Prime has built in a prediction module including all the mass vapor transmission rates (MVTR) of known packing materials, which allows you to make a better prediction by accurately selecting the type of container used in your study or to predict how the stability of your product would be in different packing materials.

## 4. Materials and Methods

### 4.1. Materials

Cristalmina^®^ (CR) (1 mL of CR: 10 mg of digluconate of chlorhexidine and q.s. of purified water) was purchased from Laboratorios SALVAT (Barcelona, Spain) (batch number: K904); digluconate of chlorhexidine (DCHX) obtained from Sigma SL (Madrid, Spain) was used as a reference solution (batch number: BCBS7878V, conc.: 20% in H_2_O); the one obtained from Cofares^®^ (batch number: 160901, conc.: 20% in H_2_O, Madrid, Spain) was used as the extemporaneously compounded solution; and p-chloroaniline (PCA) was purchased from Aldrich (Madrid, Spain) (batch number: BCBP0876V, purity: 98%). Methanol (HPLC grade, purity: ≥99.9%) was purchased from Symta SL (Madrid, Spain), while phosphoric acid (purity: 85%) was obtained from Panreac (Barcelona, Spain). The purified water was obtained through an Elix 3 Millipore purified water system (Merck, MA, USA). All other chemicals were used without further purification.

### 4.2. Methods

#### 4.2.1. Accelerated Predictive Stability (APS) Studies

The DCHX extemporaneously compounded formulation was prepared according to the Spanish National Formulary [15]; aliquots of a DCHX Cofares^®^ solution (0.25 mL) (Chlorhexidine digluconate at 20%) were withdrawn and diluted with deionized water (50 mL) to obtain a DCHX aqueous solution at 0.1%. Aliquots of CR (1 mL) were withdrawn directly from a commercialized container (Cristalmina^®^). Each aliquot (1 mL) was placed in HPLC glass vials (11.6 mm × 32 mm) and kept in test stability chambers exposed to different temperature conditions. The APS study was designed to predict stability degradation in shorter periods using extreme storage conditions (high temperatures combined with a high relative humidity). The temperatures selected were 50 °C, 60 °C, 65 °C, 70 °C, and 80 °C. Samples (5 vials for each storage condition) were analyzed at different time points: 0, 3, 7, 10, 14, 21, and 28 days. Samples were diluted, and the active pharmaceutical ingredient (API) content was analyzed by HPLC. Before the analysis, chlorhexidine aqueous solution (DCHX) aliquots were diluted with methanol (1/10). Cristalmina^®^ aliquots were diluted to 1/100 with methanol.

The HPLC analysis was carried out at room temperature (25 °C) on a modular liquid chromatograph equipped with a Jasco LC 2000-Plus series PU-1580 pump, a Jasco AS-2050 autosampler fitted to a 100 µL sampling loop, and a Jasco UV-1575 UV-visible detector. The integration of the peaks was performed with the program Borwin 1.5. A BDS Hypersil C18-RP column (250 × 4.6 mm, 5 µm) was used. The mobile phase consisted of a mixture of methanol and water containing 0.4% triethylamine (63:37, *v*:*v*). The pH was adjusted to 3.55 ± 0.02 with orthophosphoric acid measured with a Mettler Toledo MP230 GLP Research pH meter. The flow rate was 0.8 mL/min. Detection was carried out at 240 nm. The injection volume was 20 µL [16]. The method validation is described in the Supplementary Material.

#### 4.2.2. Long-Term Stability Study

The long-term study was performed with DCHX and PCA. A chlorhexidine aqueous solution at 0.1%, prepared as mentioned above, and PCA dissolved in methanol up to a 1 μg/mL concentration (RFE Mon. N°0658, Agencia Española de Medicamentos y Productos Sanitarios, 2020) were kept in different storage conditions: fridge (5 ± 3 °C), room temperature (25 °C), and at 30 °C under light exposure [15,16]. They were kept in four different packaging materials: (i) clear glass type III (Valona Pilfer-28 60 mL of capacity provided by José Mestre, Madrid, Spain), (ii) amber glass (Valona Pilfer-28 60 mL of capacity provided by José Mestre), (iii) clear plastic (Clear PET, polyethylene terephthalate, vial 60 mL of capacity, fabricated by Tecnylab, Madrid, Spain), and (iv) amber plastic (Valona Pilfer-28 PET, polyethylene terephthalate, 60 mL of capacity provided by José Mestre). At different time points (0, 3, 6, and 12, months), an aliquot was withdrawn from each container, further diluted (1/10 with methanol), and analyzed by a validated HPLC method.

According to the Spanish National Formulary, the expiration date for the extemporaneous compounding of DCHX solution is 3 months later [17,18,19], while for industrially fabricated medicines, such as CR, the shelf life is 3 years (Laboratorios SALVAT, Barcelona, Spain) [20,21].

#### 4.2.3. APS Modeling

Mathematical calculations were based on the modified Arrhenius equation (Equation (1)). The classical Arrhenius equation is good for predicting API stability from liquid dosage forms such as solutions [7]:

(1) $$Ln\;K=Ln\;A-\frac{Ea}{RT}\;$$

where *K* is the chemical reaction rate; *A* is a constant referred to as the “preexponential factor”; *Ea* is the activation energy of the reaction, typically measured in kJ/mol or Kcal/mol, that describes the “temperature sensitivity” of the drug; *R* is the universal gas constant, whose value is 8.314 J/K·mol or 1.987 cal/K·mol; and *T* is the absolute temperature expressed in Kelvin degrees. In addition, several kinetic models of degradation processes were tested to obtain a linear equation of degradation, such as the zero-order, first-order, second-order, Avrami, and diffusion equations [7,22]. In zero-order kinetics, the degradation rate is unchanged as the amount of drug substance decreases. However, it is relatively rare to find this type of reaction. Most zero-order reactions are first- or second-order reactions regarding the reactant, in which the reaction is stopped before the degradation rate begins to slow down due to the consumption of the drug. In APS studies, the specification limits for drug loss are commonly below 10%, and hence, high percentages of degradation are not relevant. Non-linear degradation models, such as the Avrami and diffusion models, are more common in pharmaceutical products due to multiple reasons, such as complex chemical degradation mechanisms in which many consecutive, competing, and reversible kinetics are involved [7,8,9].

Drug degradation was quantified along different time points to define the model’s degradation and predict its degradation profile in the long term. It was assumed at the t = 0 time point that the percent of drug degradation of DCHX and CR was zero [23]. The model with the highest *R*^2^ was selected, and the slope, which is equivalent to the degradation constant at this condition, was determined [24,25,26,27].

The degradation constant at different temperatures was plotted, and the activation energy was calculated using the Arrhenius equation [28,29,30]. From the linear equation, the degradation constant at room temperature, 25, and 30 °C was extrapolated and compared with those results obtained from the long-term stability studies at 3, 6, 9, and 12 months. Minitab software v.16 (Coventry, UK) was used to plot the predicted DCHX content from the Arrhenius equation at different points (3, 6, 9, and 12 months). An upper limit and lower limit of 110 and 90% were taken into consideration. Shelf life was calculated as the time after which you can be 95% confident that at least 50% of the response is within the specification limits. The experimental results from the long-term stored samples were also plotted and compared to the predicted values.

#### 4.2.4. Permeability Materials

The DCHX extemporaneously compounded formulation was kept in different containers, as described above, to evaluate the permeability of each packaging material. Bottles were weighed before filling and after filling with 30 mL of DCHX at different time points (0, 1, 3, 6, and 12 months) in triplicate in a precision balance (Mettler Toledo AG104 SNR 1118062020, Madrid, Spain). The evaporation rate was calculated based on the weight loss over time under the different conditions.

## 5. Conclusions

Stability studies of novel extemporaneous formulations are key to guaranteeing the efficacy and safety of medicines [31,32]. In comparison to the ICH guidelines [33,34,35], APS studies have proven to be a useful tool to predict the chemical stability of extemporaneously compounded formulations. DCHX showed different degradation kinetics than commercially available CR (Avrami vs. zero-order kinetics). This can explain the different shelf life under ICH conditions between both formulations. Predicted data for the DCHX solution were obtained from the extrapolation of the degradation rate under long-term conditions from the Arrhenius equation. The predicted shelf life for which the DCHX content was above 90% was 26.67 months under refrigerated conditions and 5.75 and 2.24 months at 25 and 30 °C, respectively. However, the Spanish National Formulary recommends a shelf life of no longer than 3 months at room temperature for DCHX solutions. Based on the predicted APS and confirmed by the experimental long-term studies, we have demonstrated that the shelf life of DCHX extemporaneously compounded formulations could be prolonged up to 6 months.

## Figures and Tables

**Table 1:** Coefficient of determination (*R*^2^) calculated for each degradation kinetic model for DCHX and CR.

Temperature (°C)	0 Order	1st Order	2nd Order	Avrami	Diffusion
Formulation	DCHX				
50	0.988	0.981	0.973	0.997	0.863
60	0.093	0.086	0.080	0.856	0.295
70	0.966	0.976	0.978	0.951	0.772
75	0.928	0.937	0.94	0.959	0.856
80	0.582	0.457	0.336	0.819	0.647
Mean *R*^2^	0.711	0.688	0.662	0.932	0.686
Formulation	CR				
50	0.884	0.877	0.870	0.419	0.630
60	0.924	0.913	0.902	0.975	0.695
70	0.820	0.809	0.796	0.874	0.823
75	0.988	0.992	0.994	0.982	0.870
80	0.949	0.911	0.856	0.884	0.998
Mean *R*^2^	0.920	0.900	0.884	0.827	0.803

**Table 2:** Calculation of activation energy (*Ea*) for DCHX and CR.

Formulation	Degradation Reaction	*E_a_* (Kcal/mol)	*R* ^2^
DCHX	Avrami	18.52 ± 2.61	0.941
CR	Zero-order	24.13 ± 3.19	0.989

**Table 3:** Permeability study in glass (clear and amber) and plastic (clear and amber) packaging materials. (T_0_ = 0 days; T_1_ = 1 month; T_2_ = 3 months; T_3_ = 6 months; T_4_ = 12 months).

Environmental Conditions	Color	Material	Weight Loss (g)				Evaporation Rate (%/day)	Average Evaporation Rate (%/day)
			(T_0_–T_1_)	(T_0_–T_2_)	(T_0_–T_3_)	(T_0_–T_4_)		
Fridge (5 ± 3 °C)	Clear	Glass	0.0033	0.0341	0.0458	0.1091	0.0003	0.0002
			0.0000	0.0028	0.0152	0.0220	0.0001	
			0.0000	0.0086	0.0140	0.0409	0.0001	
	Clear	Plastic	0.4202	0.8097	0.3032	0.8320	0.0023	0.0026
			0.5314	1.0886	0.4340	1.1458	0.0031	
			0.3858	0.7894	0.3112	0.8474	0.0023	
	Amber	Glass	0.0000	0.0000	0.0000	0.0000	0.0000	0.0000
			0.0088	0.0103	0.0000	0.0083	0.0000	
			0.0067	0.0085	0.0000	0.0068	0.0000	
	Amber	Plastic	0.5024	1.3434	0.6059	1.3992	0.0038	0.0039
			0.4990	1.2241	0.5256	1.3109	0.0036	
			0.4979	1.3292	0.6085	1.5196	0.0042	
Room Temperature (25 ± 2 °C)	Clear	Glass	0.0000	0.0152	0.0695	0.1085	0.0003	0.0002
			0.0000	0.0000	0.0481	0.0000	0.0000	
			0.0125	0.0501	0.0534	0.1416	0.0004	
	Clear	Plastic	1.2600	5.3058	2.8915	5.9977	0.0164	0.0122
			0.8258	3.5210	1.9177	3.8352	0.0105	
			0.7460	4.6935	2.0707	3.4915	0.0096	
	Amber	Glass	0.0001	0.0043	0.0059	0.0091	0.0000	0.0001
			0.0040	0.0064	0.0040	0.0109	0.0000	
			0.0394	0.0427	0.0047	0.0439	0.0001	
	Amber	Plastic	0.4046	3.6493	2.3016	4.4621	0.0122	0.0095
			0.4508	3.0884	1.8447	3.6032	0.0099	
			0.3963	2.0552	1.1622	2.3305	0.0064	
Oven (30 ± 0.5 °C)	Clear	Glass	0.0026	0.0393	0.0516	0.0946	0.0003	0.0003
			0.0252	0.0585	0.0490	0.1283	0.0004	
			0.0067	0.0317	0.0357	0.0713	0.0002	
	Clear	Plastic	0.9252	4.2107	2.3892	4.9298	0.0135	0.0142
			1.3762	4.7572	2.4185	5.9392	0.0163	
			1.3266	4.3905	2.2126	4.6557	0.0128	
	Amber	Glass	0.0009	0.0033	0.0034	0.0070	0.0000	0.0000
			0.0011	0.0039	0.0041	0.0087	0.0000	
			0.0019	0.0059	0.0061	0.0121	0.0000	
	Amber	Plastic	0.5389	8.0568	5.4851	10.8609	0.0298	0.0202
			0.5515	4.0546	2.5390	5.0225	0.0138	
			0.5709	4.8603	3.1353	6.2667	0.0172	

## Data Availability

Data are contained within the article and Supplementary Materials.

## Supplementary Materials

The following supporting information can be downloaded at https://www.mdpi.com/article/10.3390/molecules28237925/s1: Table S1: Long-term assays of DCHX content (%). Table S2: RP-C18 column dimensions and calculated parameters. Figure S1: Chromatogram of DCHX pattern (a) and PCA pattern (b) with BDS Hypersil C18 250 mm column.
