# Supplementary material for: Application of Accelerated Predictive Stability Studies in Extemporaneously Compounded Formulations of Chlorhexidine to Assess the Shelf Life
Source: Molecules. 2023 Dec 4;28(23):7925. doi: 10.3390/molecules28237925 (PMC10708018; doi:10.3390/molecules28237925)
Supplement: Supplementary file 1 [file molecules-28-07925-s001.zip › molecules-2714100-supplementary.pdf]

Supporting information

Application of Accelerated Predictive Stability Studies (APS) in extemporaneous compounding formulations of chlorhexidine to assess the shelf-life

Table S1: *Long-term assays of DCHX content (%).*

Temperature (º C)	Time (days)	Predicted content (%)	Experimental content(%) in clear glass	Experimental content (%) in clear plastic	Experimental content (%) in amber glass	Experimental content (%) in amber plastic
5	30	99.74	99.79	99.72	99.74	99.66
	60	99.48	99.38	99.19	99.3	99.19
	180	98.45	98.47	98.32	98.4	98.38
	360	96.9	96.78	96.5	96.8	97.2
25	30	98.55	99.67	99.59	99.52	99.48
	60	97.1	99.09	99.07	98.91	99.01
	180	91.29	95.46	93	93.91	92.4
	360	82.58	90.5	84.2	91.4	83.12
30	30	97.84	99.26	98.67	98.79	98.77
	60	95.69	99.08	98.76	98.96	98.55
	180	87.06	91.01	83.55	90.04	84.62
	360	74.12	80.6	71.6	78.7	79.21

Validation of chromatographic method

Different columns were analyzed to determine which one was the most suitable according to the number of plates and peak resolution (Table S2) at 8.77 min and 4.97 of retention time DCHX and PCA respectively. The BDS Hypersil C18-RP column (250 x 4.6 mm, 5 µm) showed the least interferences at 25º C with PCA degradants and exhibited an overall better resolution for both active ingredients 87.96 (DCHX) and 104.69 (PCA).

Table S2: RP-C18 column dimensions and calculated parameters.

Type of Column	Column dimensions			Column parameters			
	Length (mm)	Internal diameter (mm)	Particle size (µm)	API	Resolution	Number of plates (plates)	Pressure (MPa)
**Acclaim 300 C18 (Dionex-Thermo Fisher Scientific, California, EEUU)**	150	4.6	3	**DCHX**	57.56	359,553.211	24
				**PCA**	31.11	302,789.68	
**Nova-pak C18 (Waters Corporation, Massachusetts, EEUU)**	300	3.9	4	**DCHX**	38.98	177,903.10	28.1
				**PCA**	60.1	543,103.78	
**BDS Hypersil C18 (Hypersil-Keystone, Nueva Orleans, EEUU)**	200	4.6	5	**DCHX**	93.06	408,315.93	12
				**PCA**	64.46	663,214.63	
**BDS Hypersil C18 (Hypersil-Keystone, Nueva Orleans, EEUU)**	250	4.6	5	**DCHX**	87.96	646,521.15	12.7
				**PCA**	104.69	1,201,957.67	

a) b)

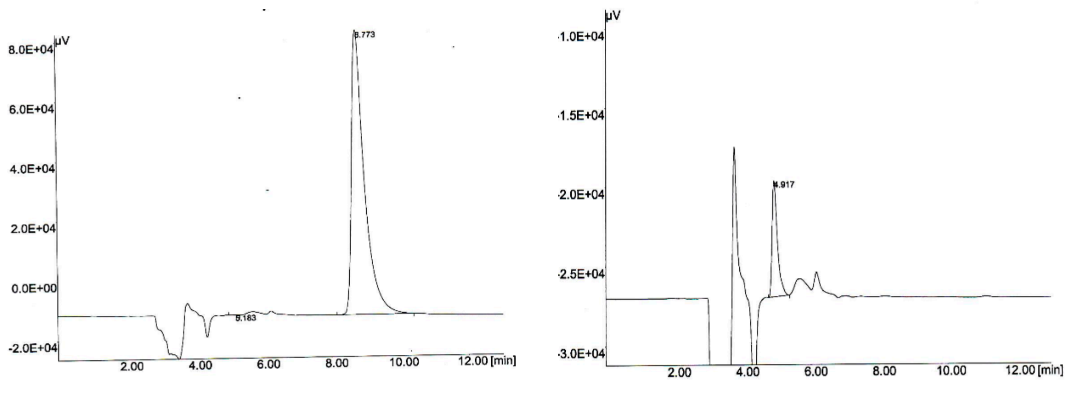

Figure S1: Chromatogram of DCHX pattern (a) and PCA pattern (b) with BDS Hypersil C18 250 mm column.
